# LESSONS FROM THE IMPLEMENTATION OF THE TRANSITIONAL CARE MODEL DURING THE COVID-19 PANDEMIC

**DOI:** 10.1093/geroni/igac059.2842

**Published:** 2022-12-20

**Authors:** Molly McHugh, Karen Hirschman, Brianna Morgan, Monica Ahrens, Alexandra Hanlon, Onome Osokpo, Mary Naylor

**Affiliations:** University of Pennsylvania, Philadelphia, Pennsylvania, United States; University of Pennsylvania, Philadelphia, Pennsylvania, United States; University of Pennsylvania, Philadelphia, Pennsylvania, United States; Virginia Polytechnic Institute and State University, Roanoke, Virginia, United States; Virginia Polytechnic Institute and State University, Roanoke, Virginia, United States; University of Pennsylvania, Philadelphia, Pennsylvania, United States; University of Pennsylvania, Philadelphia, Pennsylvania, United States

## Abstract

Multiple randomized controlled trials (RCTs) evaluating the Transitional Care Model (TCM)- an advanced practice registered nurse-led (APRN), team-based, care management strategy- have shown improved outcomes for older adults transitioning from hospital to home. In the current RCT, enrollment of older adults hospitalized with heart failure, chronic obstructive pulmonary disease, or pneumonia began in February 2020, just as the COVID-19 pandemic developed across the U.S. The COVID-19 pandemic dramatically impacted healthcare delivery across diverse local contexts. This parallel convergent mixed methods study aimed to explore the implementation of the TCM intervention as intended during Year 2 of the RCT (February 2021 to January 2022). This mixed-methods analysis presents the challenges and strategies to a key TCM component, “hospital-to-home,” which focuses on delivering in-person visits to patients, as identified through a qualitative descriptive analysis of 63 clinical team and leadership meetings combined with implementation fidelity data collected simultaneously on 188 TCM participants. In Year 2 of the trial, COVID-19-specific challenges continued, including COVID-19 exposure, policy changes, patients declining services, and limited safety equipment. Some challenges to the hospital-to-home TCM component occurred regardless of COVID-19, including patient (e.g., lack of engagement), nurse provider (e.g., TCM learning curve), and system (e.g., reduced primary care access) barriers. Collectively, these challenges resulted in lower fidelity to APRNs visit patterns during TCM delivery. Strategies to address these challenges were identified. The findings provide critical insight into how to target quality improvement strategies to improve the delivery of services, such as the TCM, from hospital to home settings.

